# Site-directed mutagenesis of Arginine282 suggests how protons and peptides are co-transported by rabbit PepT1

**DOI:** 10.1016/j.biocel.2007.10.010

**Published:** 2008

**Authors:** Myrtani Pieri, Dashiell Hall, Richard Price, Patrick Bailey, David Meredith

**Affiliations:** a Department of Physiology, Anatomy & Genetics, Le Gros Clark Building, University of Oxford, South Parks Road, Oxford OX1 3QX, UK; b School of Chemistry, University of Manchester, Oxford Road, Manchester M13 9PL, UK

**Keywords:** Epithelia, Membrane transport, SLC15a1, Nutrient absorption, Protein structure–function, Site-directed mutagenesis

## Abstract

The mammalian proton-coupled peptide transporter PepT1 is the major route of uptake for dietary nitrogen, as well as the oral absorption of a number of drugs, including β-lactam antibiotics and angiotensin-converting enzyme inhibitors. Here we have used site-directed mutagenesis to investigate further the role of conserved charged residues in transmembrane domains. Mutation of rabbit PepT1 arginine282 (R282, transmembrane domain 7) to a positive (R282K) or physiologically titratable residue (R282H), resulted in a transporter with wild-type characteristics when expressed in *Xenopus laevis* oocytes. Neutral (R282A, R282Q) or negatively charged (R282D, R282E) substitutions gave a transporter that was not stimulated by external acidification (reducing pH_out_ from 7.4 to 5.5) but transported at the same rate as the wild-type maximal rate (pH_out_ 5.5); however, only the R282E mutation was unable to concentrate substrate above the extracellular level. All of the R282 mutants showed *trans-*stimulation of efflux comparable to the wild-type, except R282E-PepT1 which was faster. A conserved negatively charged residue, aspartate341 (D341) in transmembrane domain 8 was implicated in forming a charge pair with R282, as R282E/D341R- and R282D/D341R-PepT1 had wild-type transporter characteristics. Despite their differences in ability to accumulate substrate, both R282E- and R282D-PepT1 showed an increased charge:peptide stoichiometry over the wild-type 1:1 ratio for the neutral dipeptide Gly-l-Gln, measured using two-electrode voltage clamp. This extra charge movement was linked to substrate transport, as 4-aminobenzoic acid, which binds but is not translocated, did not induce membrane potential depolarisation in R282E-expressing oocytes. A model is proposed for the substrate binding/translocation process in PepT1.

## 1 Introduction

The proton-coupled di- and tri-peptide transporter PepT1 (SLC15a1) is the major route of uptake of dietary nitrogen from the intestine, and is also important along with the higher affinity gene product PepT2 (SLC15a2) in the re-absorption of filtered peptides in the kidney (Daniel & Kottra, 2004; Meredith & Boyd, 2000). In addition, PepT1 is the route of entry of a wide class of orally bio-available pharmaceutically important compounds, including the β-lactam antibiotics, angiotensin-converting enzyme (ACE) inhibitors, antiviral and anticancer agents (Terada & Inui, 2004). Although these therapeutic compounds are not di- or tri-peptides, they are carried by virtue of their similar 3D shape to endogenous substrates, i.e. they are peptidomimetic, and modelling of the substrate-binding site from the features in common of this huge and diverse range of substrates has led to predictions concerning which parts of the PepT1 protein may be important. For example, a substrate template model has been developed by several groups (Bailey et al., 2000; Bailey et al., 2006; Biegel et al., 2005) which allows prediction of binding affinity for a potential substrate.

The rabbit PepT1 is a 707 amino acid protein, with twelve transmembrane spanning domains (TMDs) as confirmed by epitope mapping (Covitz, Amidon, & Sadee, 1998). In the absence of a crystal structure, attempts have been made to computer model the PepT1 transporter itself (Bolger et al., 1998), with site-directed mutagenesis used to test hypotheses generated by these models. One potential complication for these kind of studies is our recent report that PepT1 may form multimers in the plasma membrane (Panitsas, Boyd, & Meredith, 2006) although it is not clear how the subunits interact.

Site-directed mutagenesis has been a useful tool to identify functionally important residues in PepT1. One such residue is arginine282 in the rabbit PepT1 sequence. The mutation of arginine282 to a glutamate produced a peptide transporter (R282E-PepT1) that was no longer driven by a proton-gradient but behaved more like a facilitated peptide transporter, whilst simultaneously exhibiting peptide-gated currents that were proposed to be through a non-specific cation channel activity (Meredith, 2004). Residue 282 is located approximately halfway down the predicted transmembrane domain 7 (TMD7), and is either an arginine or a lysine in all cloned mammalian PepT1 sequences to date. The presence of a charged amino acid residue in a TMD, along with its conservation, suggested a functional role. Here, we have systematically investigated the role of arginine282 in rabbit PepT1 by making further mutations to determine the requirement for the charge and have identified an interacting residue, aspartate341, located in putative TMD8. Some of these data have been previously published in abstract form (Pieri, Boyd, & Meredith, 2004).

## 2 Materials and methods

### 2.1 Site-directed mutagenesis of the PepT1 gene

Oligonucleotides were custom synthesised (Sigma-Genosys, UK) for the following sequences (residues in bold are changed from wild-type PepT1):

- R282-PepT1 mutants forward:
  5' CGCAGATCAAGATGGTTACGxxxGTGCTGTTCCTGTACATCC 3'
  where ***xxx*** was **CAA** for R282Q, **AAG** for R282K, **GAT** for R282D, **CAT** for R282H, and **GCG** for R282A.
- D341-PepT1 mutants forward:
  5' TCCTGGTCCCCATCATGxxxGCCGTGGTGTATCC 3'
  where ***xxx*** was **CGC** for D341R.

Reverse primers for the PepT1 mutant PCR reactions were the reverse compliment of the forward primers. The site-directed PepT1 mutants were generated using the Quikchange protocol (Stratagene), and the resulting constructs confirmed by DNA sequencing (Department of Biochemistry, University of Oxford, UK).

### 2.2 cRNA synthesis and oocyte injection

PepT1 constructs were linearised with XbaI (New England Biolabs, UK) and cRNA generated by *in vitro* transcription (T7 mMessage mMachine, Ambion, Cambridgeshire, UK). *X. laevis* oocytes were obtained under MS222 anaesthesia (0.2%, w/v) in accordance with the UK Animals (Scientific Procedures) Act, 1986, and maintained at 18 °C in modified Barth's medium (88 mM NaCl, 1 mM KCl, 0.82 mM MgSO_4_, 2.4 mM NaHCO_3_, 0.42 mM CaCl_2_, 10 mM Hepes, 5 mM sodium pyruvate, 50 μg ml^−1^ gentamicin (Fluka, Poole, UK), adjusted to pH 7.6 with 1 M NaOH). Transport measurements were performed at least 72 h after micro-injection of oocytes with 27nl cRNA (1 μg/μl), with medium changed daily.

### 2.3 Transport experiments

Zero-*trans* uptake of [^3^H]-d-Phe-l-Gln (17.4 Ci/mmole, custom synthesised, Cambridge Research Biochemicals, Stockton-on-Tees, UK) was performed as previously described (Meredith, 2004). Briefly, 5 oocytes were incubated in 100 μl of uptake medium (95 mM NaCl, 2 mM KCl, 1 mM CaCl_2_, 0.42 mM MgCl_2_, 10 mM Tris/Hepes pH 7.4 or Tris/Mes pH 5.5) with tracer (0.4 μM) [^3^H]-d-Phe-l-Gln. After incubation, the oocytes were washed sequentially five times in 1 ml of ice-cold 120 mM NaCl solution, lysed individually with 100 μl 2% (w/v) SDS and liquid scintillation counted. As a control non-injected oocytes were also incubated in uptake medium with [^3^H]-d-Phe-l-Gln as above.

The affinity of wild-type and mutant PepT1 were assessed by competition studies with 0.4 μM [^3^H]-d-Phe-l-Gln and Gly-l-Gln present in the uptake medium in concentrations from 0 to 2 mM using the protocol above, and the *K*_i_ calculated using the method of Deves and Boyd (1989).

Efflux studies were performed as previously described (Meredith, 2004), with the exception that the extracellular *trans-*stimulant Gly-l-Gln was used at 5 mM, and an efflux time-course was performed.

Diethylpyrocarbonate (DEPC) inhibition of PepT1 was performed using a similar protocol to that of Terada, Saito, and Inui (1998). Briefly, PepT1 oocytes were preincubated with 1 mM DEPC for 15 min at pH_out_ 5.5 in the absence or presence of the PepT1 substrates Gly-l-Gln, *N*-Acetyl-Phe (Meredith et al., 2000), and l-Ala-Tyramine (custom synthesised) and the non-substrate Tyr (all 5 mM). The oocytes were then washed in uptake medium before uptake assays were performed as detailed above.

### 2.4 Electrophysiology

Measurements of membrane potential were made by impaling oocytes with a single glass microelectrode (Intra 767 amplifier, WPI, Stevenage, Hertfordshire, UK) perfused in uptake medium (as above) with or without 0.4 μM d-Phe-l-Gln (synthesised in house), 0.6 mM Gly-l-Gln (Sigma, Poole, UK) or 10 mM 4-aminobenzoic acid (4-AMBA, Sigma). Two-electrode voltage clamp (TEVC) was performed by placing oocytes in a 0.1 ml recording chamber and perfusing with uptake solution (pH 5.5 or 7.4) at a rate of 15 ml/min. Oocytes were impaled by two agarose-cushioned microelectrodes filled with 3 M KCl (0.5–2.0 MΩ) and voltage-clamped at −60 mV using a Geneclamp 500B amplifier and PCLAMP 8.1 software (Axon Instruments, CA, USA). The holding potential was stepped from −60 mV over the range of −150 to +50 mV in 10 mV steps, each pulse lasting 100 ms, and returning to −60 mV in between test voltage pulses. Typically traces were filtered at 1 kHz during recording and digitized at 0.5–5 kHz using the DigiData 1200 interface (Axon Instruments, CA, USA). All experiments were carried out at room temperature.

### 2.5 Data analysis

All data are expressed as mean ± S.E.M., except for Fig. 6 where the error bars represent the maximum range of stoichiometry values when taking into account the errors for the uptake data and the currents.

In order to calculate the apparent charge:substrate stoichiometry, the peptide induced current that was measured by two-electrode voltage clamp at the oocyte resting membrane potential was divided by the radiolabelled dipeptide uptake in oocytes from the same preparation. This value was normalised to 1:1 for wild-type PepT1, the accepted stoichiometry for a neutral dipeptide (Fei et al., 1994; Steel et al., 1997).

### 2.6 Statistical analysis

Statistical analyses were performed using one-way ANOVA with differences considered significant if *p* < 0.05 when data were compared to the wild-type control, as detailed in the text and/or figure legend.

## 3 Results

### 3.1 pH dependence of d-Phe-l-Gln uptake into R282 mutants

**Fig. 1:**
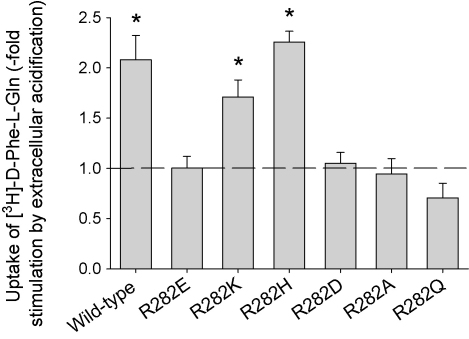
pH dependence of initial rate [^3^H]-d-Phe-l-Gln uptake by *Xenopus* oocytes expressing R282 mutants of rabbit PepT1, expressed as -fold stimulation by extracellular acidification (pH_out_ 7.4 to 5.5), the dotted line showing where there is no stimulation (*n* ≥ 3 oocyte preparations with at least 5 oocytes per data point for each, except for R282Q-PepT1 where *n* = 2; ^*^p < 0.05 for stimulation by external acidification, one-way ANOVA).

**Fig. 2:**
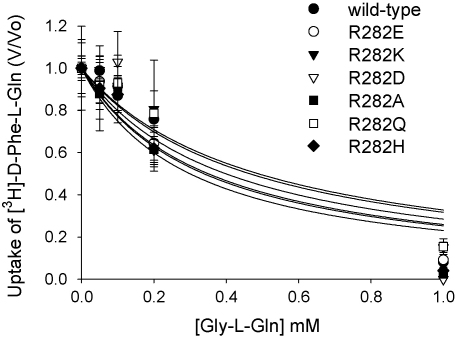
Inhibition of [^3^H]-d-Phe-l-Gln uptake by increasing concentrations of Gly-l-Gln (0–1 mM) into *Xenopus* oocytes expressing either the various R282 mutants or wild-type PepT1 at pH_out_ 5.5. *K*_i_ values were wild-type PepT1 0.39 ± 0.22, R282E 0.35 ± 0.17, R282K 0.48 ± 0.26, R282D 0.34 ± 0.27, R282A 0.30 ± 0.17, R282Q 0.46 ± 0.22 and R282H 0.30 ± 0.15 mM. *n* = 5 oocytes per data point.

Fig. 1 shows the pH dependence of d-Phe-l-Gln uptake into oocytes expressing mutant PepT1 transporters where R282 has been changed into number of amino acid residues. Of the residues tested, R282K- and R282H-PepT1 behaved like the wild-type PepT1, in that the initial rate of uptake (1 h incubation time) of dipeptide was significantly faster (*p* < 0.05, one-way ANOVA) at an external pH of 5.5 than at 7.4. The other amino acid substitutions tested, R282E- (as previously reported, Meredith, 2004), R282D-, R282A- and R282Q-PepT1 all gave the same initial rate of uptake at pH 5.5 and 7.4, indicating that transport by these mutants is not stimulated by an inwardly directed proton gradient. These changes cannot be ascribed to changes in the affinity of the mutant PepT1 proteins for their substrate, as the *K*_i_ of Gly-l-Gln inhibiting 0.4 μM [^3^H]-d-Phe-l-Gln was unchanged in the mutants (Fig. 2).

### 3.2 Can the R282 mutant PepT1 transporters concentrate substrates?

**Fig. 3:**
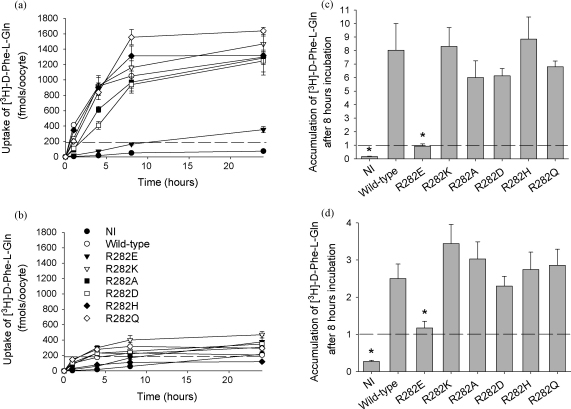
Accumulation of [^3^H]-d-Phe-l-Gln above the extracellular concentration by *Xenopus* oocytes expressing R282 mutants of rabbit PepT1. Fig. 3a and b shows representative time-course experiments at pH_out_ 5.5 and 7.4, respectively. Fig. 3c and d shows the accumulation ratios at pH_out_ 5.5 and 7.4 after 8 h incubation, respectively. The dotted line represents the equilibrium value if the volume of an oocyte is assumed to be 1 μl (^*^p < 0.05, one-way ANOVA). For R282E-PepT1 at pH_out_ 7.4, the same level of accumulation was reached after 24 h incubation (2.3 ± 0.4-fold, *p* > 0.05, one-way ANOVA, *n* = 5 oocyte preparations with 5 oocytes per data point for each) as for the wild-type and the other mutants (see Section 3 for further details). NI = non-injected control oocytes.

An earlier finding was that, unlike the wild-type, the R282E-PepT1 mutant was unable to concentrate substrate even in the presence of an inwardly directed proton gradient (Meredith, 2004). The ability to concentrate substrate was therefore tested for the other R282 mutants (Fig. 3a and b shows representative time-course experiments at pH_out_ 5.5 and 7.4, respectively), and the mean accumulation levels are shown for 8 h uptakes in Fig. 3c for pH_out_ 5.5. In contrast to R282E-PepT1, all were found to be able to concentrate peptide well above the equilibrium level when the external pH was 5.5 (an oocyte was assumed to have a volume of 1 μl, Petersen & Berridge, 1996; Yao & Tsien, 1997). In the absence of the proton driving force (pH_out_ 7.4, Fig. 3d) a similar level of intracellular d-Phe-l-Gln concentration was reached by all the mutant PepT1 transporters, including R282E-PepT1 if the incubation time was increased to 24 h (accumulation of 2.3 ± 0.4-fold compared to 3.1 ± 0.5-fold for wild-type PepT1). Although at 24 h incubation times we observed that cell survival can be a limiting factor, there was no statistical increase in the accumulation for the wild-type PepT1 at pH_out_ 5.5 or 7.4 between 8 and 24 h incubations, nor between 8 and 24 h for the wild-type PepT1 at pH_out_ 7.4 (8.0 ± 1.4 vs. 8.3 ± 1.0, 2.5 ± 0.5 vs. 3.1 ± 0.5 and 1.0 ± 0.4 vs. 1.5 ± 0.3 respectively, all *p* > 0.05, one-way ANOVA).

### 3.3 Rates of d-Phe-l-Gln efflux from R282 mutants

**Fig. 4:**
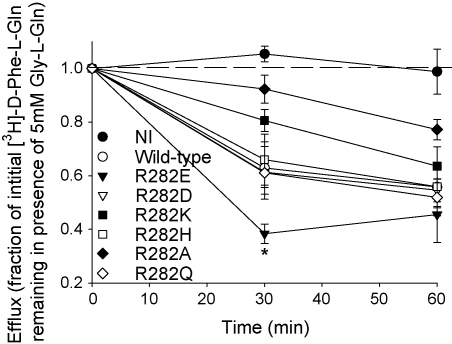
Efflux of intracellular [^3^H]-d-Phe-l-Gln in the presence of 5 mM Gly-l-Gln expressed as a fraction of that seen in the absence of *trans-*stimulant for non-injected control oocytes (NI), wild-type and R282 mutant PepT1 expressing *Xenopus* oocytes. Efflux by R282E-PepT1 at 30 min was significantly faster than for the wild-type and other R282 mutant PepT1s (^*^p < 0.05, one-way ANOVA), whilst efflux from all of the PepT1 expressing oocytes was faster than that for non-injected. After 60 min, the effluxes from all the PepT1 expressing oocytes were not significantly different, whilst significantly faster than for non-injected controls (*p* < 0.05, one-way ANOVA). *n* = 5 oocytes per data point.

Fig. 4 shows the rates of efflux of [^3^H]-d-Phe-l-Gln from oocytes expressing either wild-type PepT1, R282 PepT1 mutants or non-injected controls. All of the PepT1 constructs showed a significantly faster efflux than the non-injected oocytes (one-way ANOVA, *p* < 0.05), whilst R282E-PepT1 was significantly faster than the wild-type (one-way ANOVA, *p* < 0.05) as previously described (Meredith, 2004). Interestingly, R282E-PepT1 showed a faster efflux than all the other R282 mutants (one-way ANOVA, *p* < 0.05), which were not significantly different to the wild-type (*p* = 0.14).

### 3.4 Apparent transport stoichiometry (charge to substrate) using two-electrode voltage clamp

**Fig. 5:**
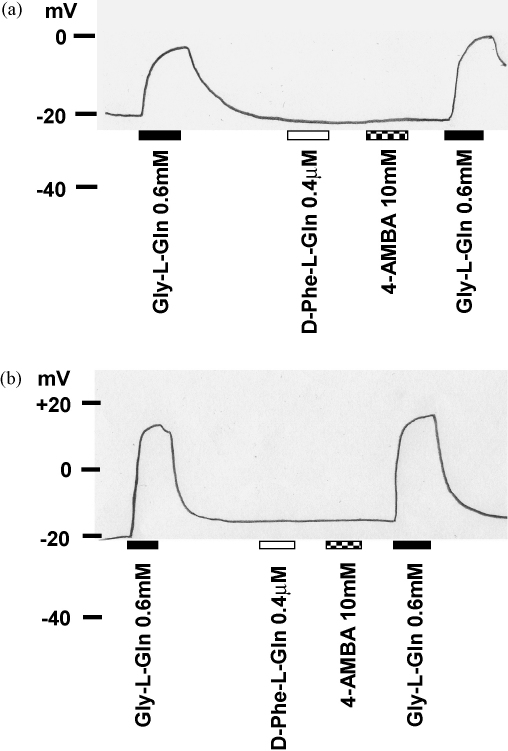
Representative trace of membrane potential from an oocyte expressing wild-type PepT1 (Fig. 5a) or R282E-PepT1 (Fig. 5b) in the absence or presence of Gly-l-Gln, d-Phe-l-Gln and 4-AMBA (0.6 mM, 0.4 μM and 10 mM respectively, for 1 min each), at pH_out_ 5.5. Non-injected control oocytes showed no response (data not shown), with similar responses seen for at least 3 oocytes.

**Fig. 6:**
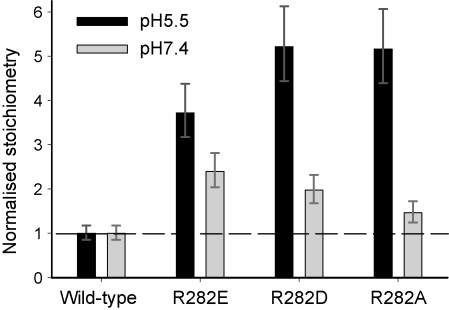
Charge:peptide stoichiometry of uptake of Gly-l-Gln in *Xenopus* oocytes expressing R282E-, R282D- or R282A-PepT1, normalised to that of the wild-type PepT1 being 1:1 as previously reported (Fei et al., 1994; Steel et al., 1997). The average error on the current measurements was 13 ± 3.8% (*n* = 3 oocytes per mutant) and on the uptakes 4.4 ± 0.7% (*n* = 5 oocytes per mutant); the error bars on the figure represent the extremes of the stoichiometry that would be calculated using these error values.

As well as uptake being pH independent and non-concentrative, the electrophysiological characteristics of R282E-PepT1 were strikingly different to the wild-type (Meredith, 2004). Since R282D-PepT1 is similarly pH independent but does accumulate substrate, the apparent proton to peptide stoichiometry was examined, where the uptake of 0.4 μM d-Phe-l-Gln was compared to the peptide-induced current in the same batch of oocytes. The current at the oocyte resting membrane potential (−27.0 ± 1.1 mV at pH_out_ 5.5, and −36.8 ± 2.2 mV at pH_out_ 7.4, *n* = 12) was taken to represent the membrane potential under uptake conditions, as the addition of 0.4 μM substrate does not produce a detectable change in membrane potential (Fig. 5). The ratio of proton to neutral dipeptide co-transported through wild-type PepT1 is 1:1 (Fei et al., 1994; Steel et al., 1997), yet in R282E-, R282D- and R282A-PepT1 the apparent stoichiometry is substantially higher (4, 5 and 5 respectively at pH_out_ 5.5, Fig. 6).

### 3.5 Is the extra current measured dependent on substrate transport?

In an attempt to see if the extra current carried by R282E-PepT1 was dependent on substrate translocation rather than simply substrate binding, the non-translocated PepT1 substrate 4-aminobenzoic acid (4-AMBA, Darcel, Liou, Tome, & Raybould, 2005; Meredith et al., 1998) was used. As found for wild-type PepT1, 4-AMBA failed to induce a depolarisation in R282E-PepT1 oocytes at 10 mM, three times its *K*_i_ (Meredith et al., 2000), in contrast to the known transported substrate Gly-l-Gln, also at three times *K*_i_ (Fig. 5).

### 3.6 Identification of an interacting residue for R282

**Fig. 7:**
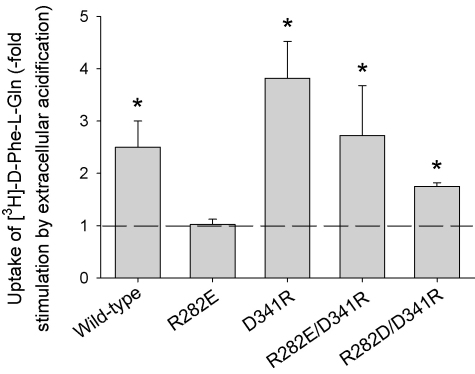
pH dependence of initial rate [^3^H]-d-Phe-l-Gln uptake into oocytes expressing wild-type, R282E-, D341R-, R282E/D341R- and R282D/D341R-PepT1 expressed as -fold stimulation by extracellular acidification (pH_out_ 7.4–5.5), the dotted line showing where there is no stimulation (^*^p < 0.05 for the -fold stimulation being greater than one, one-way ANOVA, *n* ≥ 3 oocyte preparations).

The conservation of a positively charged amino acid in a transmembrane domain (TMD7), and the results above, strongly suggested that the presence of a positive charged residue was necessary for wild-type-like PepT1 transport function. In TMD8, predicted to be at approximately the same level in the membrane, there is a conserved aspartate (D341), and it was an appealing hypothesis that the two oppositely charged side chains might be forming a charge pair. To test this, double mutants were made, R282E/D341R- and R282D/D341R-PepT1, to swap the charges over. As can be seen in Fig. 7, these double mutants showed the same pH dependence of influx as the wild-type transporter, providing strong evidence to support the hypothesis. Both R282E/D341R- and R282D/D341R-PepT1 were also able to concentrate substrate like the wild-type (data not shown).

### 3.7 Diethylpyrocarbonate inhibition of PepT1 function

**Fig. 8:**
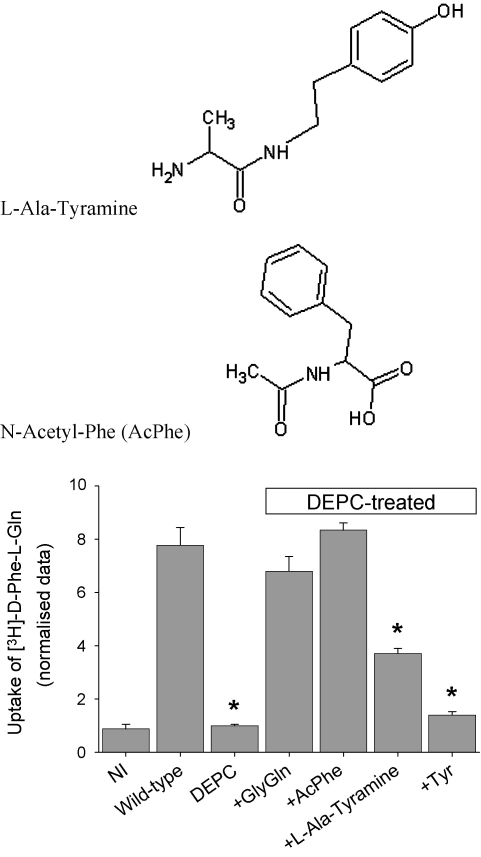
Protection of PepT1 from diethylpyrocarbonate (DEPC) inhibition (1 mM, 15 min incubation) by Gly-l-Gln (GlyGln), *N*-Acetyl-Phe (AcPhe) and l-Ala-Tyramine, but not Tyr (all present at 5 mM during DEPC incubation period). [^3^H]-d-Phe-l-Gln uptake was measured over 1 h. ^*^p < 0.05 when compared to wild-type PepT1 (one-way ANOVA, 5 oocytes per data point, *n* = 3 oocyte preparations). NI = non-injected control oocytes.

Preincubation of wild-type PepT1-expressing oocytes with 1 mM diethylpyrocarbonate (DEPC) for 15 min completely inhibited the PepT1 mediated dipeptide uptake measured over 1 h, as shown in Fig. 8. This inhibition by DEPC was largely prevented by the presence of the known PepT1 substrates Gly-l-Gln and *N*-Acetyl-Phe (Meredith et al., 2000), but not by the amino acid non-substrate Tyr. Interestingly, despite being a good PepT1 substrate (*K*_i_ approximately 0.1 mM, data not shown), a modified peptide lacking a carboxyl terminus (l-Ala-Tyramine) only partially prevented DEPC inhibition (Fig. 8).

## 4 Discussion

The R282K mutation of rabbit PepT1 is not only the most conservative one regarding the charge, but in a number of species, including dog, rat and mouse, lysine is the naturally occurring residue at this position. Therefore it was not surprising to find that this mutant behaves like the wild-type rabbit PepT1 (pig, sheep, rhesus and crab-eating monkeys and human also have R282). The finding that R282H also behaved like the wild-type was interesting, as histidine has a side-chain that can be titrated over the range used in the experiments (p*K* of ∼6 in free solution). Our findings could be interpreted in several ways, including the possibility that only at pH_out_ 5.5 is there the formation of a positive charge by side-chain titration that gives a stimulation of uptake over that seen at pH_out_ 7.4. A second possibility is that the protein environment surrounding H282 is such that its side chain p*K* is shifted away from 6 and it is therefore always protonated, and thus behaves more like an arginine or lysine. This effect has been shown for example in the enzyme protein tyrosine kinase (Tishmack, Bashford, Harms, & Van Etten, 1997), where histidine residues had a p*K* as high as 9.2 when analysed by NMR.

In the original study on R282E-PepT1, it was concluded that the uptake of peptide by the mutant transporter was uncoupled from the movement of protons, and that in addition to acting as a facilitated peptide transporter, R282E-PepT1 also displayed a peptide-gated non-specific cation conductance (Meredith, 2004). However, it is possible that this conclusion needs updating in the light of the current findings that there are R282 mutants that, like R282E-PepT1, are not pH stimulated, yet are still able to accumulate substrate above equilibrium when an inwardly directed proton gradient is imposed. For a transporter to be able to accumulate substrate above equilibrium, an energy source must be involved, in this case the proton electrochemical gradient. Therefore, the mutant PepT1 proteins that can accumulate substrate but do not show pH stimulation (R282A and R282D) must still be coupled to the movement of protons down their electrochemical gradient. The lack of pH stimulation could be attributed to the fact that during the transport cycle these specific mutants have a different rate limiting step to the wild-type, and that for these mutants that step is not pH dependent. It has already been reported that R282E-PepT1 has a faster rate of efflux than wild-type, consistent with an uncoupling of peptide uptake from the proton driving force (Meredith, 2004); the finding here that the rates of efflux for the other R282 mutants are not different from that of the wild-type is in agreement with the hypothesis that they are still proton-coupled, as shown by their ability to accumulate substrate above the extracellular concentration at pH_out_ 5.5 but not 7.4.

The simplest hypothesis was that in R282E-PepT1, the extra inward charge movement associated with peptide uptake collapsed the membrane potential, which is known to be the major driving force for proton-coupled peptide uptake (Temple & Boyd, 1998), hence the apparent lack of substrate accumulation. Therefore by extension one would expect R282D- and R282A-PepT1 to have the same charge coupling as the wild-type, as they too accumulate substrate, but this was not the case: both R282E-, R282D- and R282A-PepT1 showed the same increased charge:peptide apparent stoichiometry, i.e. substantially larger than the wild-type. This stoichiometry itself showed pH dependence, with a lower value of around 2 for the mutants at pH 7.4, suggesting that the current is either carried by protons or is a pH-sensitive phenomenon. There was no difference in the uptake in the absence or presence of sodium, either for R282E-PepT1 (Meredith, 2004) or R282D-PepT1 (data not shown).

The finding that the R282E/D341R- and R282D/D341R-PepT1 double mutants (the latter being a charge pair reversal of the naturally occurring residues in rabbit PepT1) had the same characteristics as the wild-type protein strongly suggests that these two residues do interact in the 3D protein (Pieri et al., 2004) as previously proposed (Meredith, 2004). During the preparation of this manuscript, Kulkarni et al. (2007) reported findings consistent with R282 and D341 forming a charge pair in human PepT1. The initial hypothesis that in R282E-PepT1 repulsion between E282 and D341 allowed the movement of extra ions through the protein when a peptide is transported was not supported however by the finding that the single D341R mutant also behaved like the wild-type, as one might have thought that R282 and R341 would repel in much the same way as E282 and D341. That D341R-PepT1 behaved like wild-type suggests that although R282 and D341 seem to form a charge pair, in rabbit PepT1 residue 341 being negatively charged is not crucial to PepT1 function; interestingly, in human PepT1 the D341R single mutant had reduced function (Kulkarni et al., 2007). The reason for this difference between rabbit and human PepT1 is not clear. The observation that the non-transported PepT1 substrate 4-AMBA did not induce a depolarisation in R282E-PepT1-expressing oocytes clearly shows that the charge movement is linked to substrate binding and translocation and not to binding alone. One explanation for the increase in proton–peptide stoichiometry is that in wild-type PepT1 the presence of a positively charged residue deep in the binding pocket at position 282 repels proton movement through the transporter protein during the translocation step. The data in Fig. 6 are consistent with this, as the apparent stoichiometry is lower at pH_out_ 7.4 than it is at pH_out_ 5.5, indicating that the proton electrochemical gradient is involved.

In the case of mutations where R282 was replaced with a non-positively charged amino acid, the rate-limiting step of the transport cycle must be insensitive to extracellular pH, hence the lack of stimulation when the pH_out_ is dropped from 7.4 to 5.5. Kinetic analysis of peptide transport by PepT1 in rat renal brush border membrane vesicles (Temple & Boyd, 1998) showed that at pH_out_7.4/pH_in_7.4, it was the protonation of the carrier protein that was the rate-limiting step (Temple, Bailey, Bronk, & Boyd, 1996), whereas at pH_out_5.5/pH_in_7.4 it was the return of the unloaded carrier. The rate of peptide uptake by R282E-PepT1 (corrected for protein expression in the intact oocyte plasma membrane by luminometry, Panitsas et al., 2006) is the same as for the wild-type at pH_out_ 5.5 (data not shown), but, unlike for the wild-type, is not slower at pH_out_ 7.4. This indicates that the mutations to arginine282 that abolish pH sensitivity (R to E, D, A, or Q) are affecting the rate limiting step, so when pH_out_ is 7.4 the rate limiting step has the same magnitude as that at pH_out_ 5.5, hence the lack of sensitivity to changing pH_out_. The rate limiting protonation of the outward-facing carrier protein at pH_out_ 7.4, the first step in the transport cycle (Temple et al., 1996), was proposed by us (Bailey et al., 2000; Meredith & Boyd, 1995) and others (e.g. Steel et al., 1997; Uchiyama, Kulkarni, Davies, & Lee, 2003) to be protonation of histidine57 (H57). In the R282-PepT1 mutants, except for R282K and R282H, the lack of a positively charged residue might result in a conformational change of the protein that changes the local environment and increases the p*K*_a_ of H57, such that it is more easily protonated at a higher pH_out_. Thus at pH_out_ 7.4 the rate limiting step is no longer the protonation of H57, but the return of the unloaded carrier, as it is at pH_out_ 5.5, hence the similar transport rates at pH_out_ 5.5 and 7.4.

**Fig. 9:**
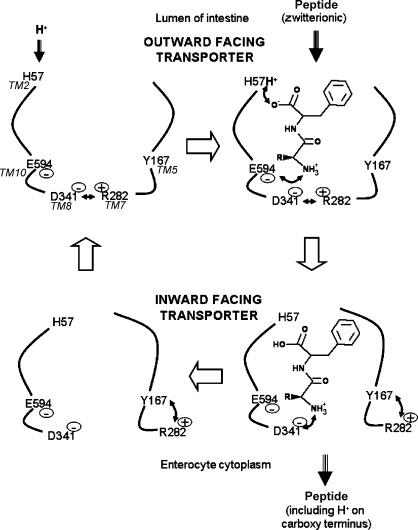
Model for PepT1 substrate binding and translocation. Arrows represent salt bridges or hydrogen bonds between residues.

A model for how proton–peptide transport might occur is shown in Fig. 9: the empty PepT1 is primed by protonation of H57, followed by the binding of a zwitterionic peptide, with the N-terminal co-ordinated at E595 and the C-terminal at H57-H^+^ (Meredith et al., 2000). Although the binding of the substrate C-terminal to His57-H^+^ is in disagreement with the conclusion of Terada et al. (1998), it is supported by the finding that *N*-Acetyl-Phe, a known PepT1 substrate which does not have a free amino terminal (Meredith et al., 2000), can protect PepT1 against inhibition by DEPC. Additionally, the finding that significant DEPC inhibition is still evident when oocytes were co-incubated with the PepT1 substrate l-Ala-Tyramine which lacks a carboxyl terminus further adds to the notion that H57-H^+^ binds the carboxyl terminus of the substrate (Fig. 8). H57-H^+^ then donates its proton to the C-terminal carboxyl group, and the transporter undergoes a conformational change that leads to the breaking of the salt bridge between R282 and D341, re-orientating the binding site to be inward facing as simultaneously the protonated N-terminal of the substrate binds to D341, neutralising the charge, and the R282 charge is stabilised by Y167 (the chemical properties of this tyrosine have been shown to be essential, Yeung et al., 1998). The peptide molecule is then released into the cytoplasm, whereby it returns to the zwitterionic state by releasing the proton from the carboxyl terminal. The transporter then undergoes the reverse conformational change to re-orientate the binding site to outward facing, and R282 reforms the salt bridge with D341.

As R282E-PepT1 cannot accumulate substrate, the implication must be that the movement of peptide is no longer coupled to the movement of the protons, whereas in all the other mutants coupling must be maintained. If for R282E-PepT1 the p*K*_a_ of H57 was raised to the point that it was no longer favourable for it to donate its proton to the carboxyl terminus of the substrate, then peptide transport would no longer be proton-coupled, and this would explain the failure of R282E-PepT1 to accumulate substrate. Intriguingly, it can be seen in Fig. 6 that both R282D- and R282A-PepT1 appear to carry approximately one more charge per substrate peptide than R282E-PepT1, which is consistent with the hypothesis that one of the charges carried is coupled with the substrate.

In conclusion, the arginine at position 282 in rabbit PepT1 plays an intriguing role in the function of the transporter, with mutations to different residues revealing that a positive residue is required for pH dependence, whilst only R282E-PepT1 cannot concentrate substrate above equilibrium; this is despite other mutations, most notably R282D-PepT1, having a similarly increased charge:peptide stoichiometry. As previously proposed, R282 (TMD7) forms a charge pair with D341 (TMD8), with R282E/D341R-PepT1 showing normal transport characteristics. Further biological testing or a crystal structure of PepT1 will be required to establish the validity of the model proposed.
